# Adding Value of MRI over CT in Predicting Peritoneal Cancer Index and Completeness of Cytoreduction

**DOI:** 10.3390/diagnostics11040674

**Published:** 2021-04-08

**Authors:** Chia-Ni Lin, Weh-Shih Huang, Tzu-Hao Huang, Chao-Yu Chen, Cheng-Yi Huang, Ting-Yao Wang, Yu-San Liao, Li-Wen Lee

**Affiliations:** 1 Department of Diagnostic Radiology, Chang Gung Memorial Hospital, Chiayi 613016, Taiwan; lcn6979@cgmh.org.tw (C.-N.L.); mm601200@gmail.com (Y.-S.L.); 2 Division of Colorectal Surgery, Department of Surgery, Chang Gung Memorial Hospital, Chiayi 613016, Taiwan; wen1204@cgmh.org.tw (W.-S.H.); bluesky@cgmh.org.tw (C.-Y.H.); 3 Division of General Surgery, Department of Surgery, Chang Gung Memorial Hospital, Chiayi 613016, Taiwan; tambobo8916@gmail.com; 4 Department of Obstetrics and Gynecology, Chang Gung Memorial Hospital, Chiayi 613016, Taiwan; b9002031@cgmh.org.tw; 5 Division of Hematology and Oncology, Department of Internal Medicine, Chang Gung Memorial Hospital, Chiayi 613016, Taiwan; tywang.onco@gmail.com

**Keywords:** hyperthermic intraperitoneal chemotherapies, peritoneal carcinomatoses, cytoreductive surgery

## Abstract

Background: This study aimed to investigate the adding value of MRI over CT for preoperative cytoreductive surgery with hyperthermic intraperitoneal chemotherapies (CRS/HIPEC). Methods: Imaging and intraoperative peritoneal cancer index (PCI) were calculated in 62 patients with peritoneal metastasis. Predictive models for the completeness of cytoreductive score using PCI data were established using decision tree algorithms. Results: In gastric cancer patients, a large discrepancy and poor agreement was appreciated between CT and surgical PCI, and a nonsignificant difference was noted between MRI and surgical PCI. In colon cancer patients, a better agreement and higher correlation with a smaller error was observed in PCI score using MRI than in that using CT. However, the addition of MRI to CT was limited for appendiceal and ovarian cancer patients. For predicting incomplete cytoreduction, CT models yielded inadequate accuracy while MRI models were more accurate with fair discrimination ability. Conclusions: CT was suitable for estimating PCI and surgery outcome in appendiceal and ovarian cancer patients, while further MRI in addition to CT was recommended for colon and gastric cancer patients. However, for classifying patients with peritoneal carcinomatosis into complete and incomplete cytoreduction, MRI was more effective than CT.

## 1. Introduction

Peritoneal metastasis is defined as cancer that has spread or metastasized to the peritoneal cavity. Traditionally, it was considered as an advanced disease with a poor prognosis [1]. Since the 1980s, surgical oncologists have developed an approach combining cytoreductive surgery (CRS) and hyperthermic intraperitoneal chemotherapy (HIPEC) for patients with peritoneal metastasis [2]. CRS is a surgical procedure that removes macroscopic disease from the abdominal cavity, while HIPEC is an intraoperative procedure that infuses and circulates heated chemotherapeutic agents into the peritoneal cavity to treat microscopic residual disease [3]. With advanced medical technology, CRS/HIPEC has become an important and promising treatment option for peritoneal metastasis [4], but is associated with a significant risk of morbidity and mortality [5,6].

Notably, it is essential to carefully identify the patients benefiting from treatments, as the inaccurate diagnosis of the extent of metastasis results in futile CRS/HIPEC [7,8]. The most common approach for quantifying the size and distribution of peritoneal tumors is the Sugarbaker’s Peritoneal Cancer Index (PCI) [9]. The PCI scoring system divides the abdominopelvic cavity into 13 separate regions, and each region is scored 0–3 points as follows: 0 point, the absence of tumor; 1 point, tumors < 0.5 cm in diameter; 2 points, tumors 0.5–5 cm in diameter; 3 points, tumors > 5 cm in diameter. Residual tumors after CRS are assessed by the scores of completeness of cytoreduction (CC) [10]. A CC-0 resection is defined as no visible tumor following CRS, CC-1 resection indicates persisting tumors < 2.5 mm, CC-2 corresponds to visible tumors between 2.5 mm and 2.5 cm, and CC-3 indicates residual tumors > 2.5 cm.

In the multidisciplinary team of CRS/HIPEC, the responsibilities of a radiologist are to accurately interpret the preoperative disease status and make recommendations for further imaging tests. The diagnostic performance of peritoneal metastasis depends on radiologist experience, imaging modality, lesion morphology, location, and histology. Even for experienced radiologists, image interpretation of peritoneal metastasis remains challenging. Using multidetector CT, the sensitivity improved from 11% with lesion size <0.5 cm to 94% with lesion size >5 cm for detecting peritoneal tumor due to technical limitations [11]. Even for large tumors, missed diagnoses still occur if the tumor is very thin coating on the parietal or visceral peritoneum rather than nodular [12]. Another important factor is the histological types of peritoneal metastasis. On CT, it is difficult to distinguish mucinous ascites from pseudomyxoma peritonei and depict tumor invasion of the mesentery [13]. Providing better soft tissue contrast, contrast-enhanced MRI is optimal to depict these lesions. However, MRI is more susceptible to motion artifacts than CT because of the long scanning time.

CT is the most frequently used imaging modality to investigate patients with the suspicion of peritoneal carcinomatosis. However, MRI may be superior to CT for the determination of preoperative PCI score under certain circumstances. On MRI combined with diffusion-weighted imaging (DWI), the sensitivity and specificity was 90% and 95.5%, respectively [14]. Unfortunately, there is no universal consensus on the radiological studies for patients who underwent CRS/HIPEC assessment. Therefore, radiologists are presented with new challenges to recommend individually tailored imaging studies for selected patients undergoing CRS/HIPEC while taking into account the benefit and cost of healthcare [6].

In this study, the correlations between imaging PCI by CT (CT-PCI) and MRI (MRI-PCI) and intraoperative PCI (OP-PCI) were explored. The performance of imaging and intraoperative PCI for predicting the completeness of cytoreduction for patients with peritoneal metastasis from appendiceal, colon, ovarian, and gastric origins was also assessed using decision tree models.

## 2. Materials and Methods

### 2.1. Study Design

This was a prospective, single-institution study of CT and MRI versus the surgical standard of reference for predicting surgical outcome in patients with peritoneal metastasis. All subjects gave their written informed consents for inclusion before they participated in the study. The study was conducted in accordance with the Declaration of Helsinki, and the protocol was approved by the Institutional Review Board of Chang Gung Medical Foundation (No. 01700545B0 and 201701365A3).

### 2.2. Study Population

Patients scheduled for CRS/HIPEC at the institution and who had preoperative abdominopelvic CT scans were invited. Exclusion criteria were patients with age < 20 years, impaired renal function (eGFR < 45 mL/min/1.73 m^2^), and with MRI contraindications. Contrast-enhanced MRI studies were arranged for participants meeting the criteria before surgery. From January 2018 to October 2020, a total of 81 patients agreed to participate in the study and were subjected to CT and MRI studies. From this, 18 patients were excluded due to no subsequent diagnostic or exploratory laparotomy; 1 patient was excluded due to severe motion artifacts of MRI images. Of the 62 patients included, the primary tumors arise from appendix (*n* = 6), colon (*n* = 25), ovary (*n* = 20), and stomach (*n* = 11). Preoperative imaging PCI including CT-PCI and MRI-PCI and OP-PCI were obtained from all included patients for further analysis.

### 2.3. Imaging Studies

CT scans of the abdomen and pelvis were performed as part of routine practice on three multidetector CT scanners: Somatom Sensation 64 (Siemens Healthcare, Erlangen, Germany), Aquilion 64 (Toshiba Medical System, Tokyo, Japan), and Aquilion ONE (Canon Medical Systems, Otawara, Japan). Patients were fasted for at least 4 h before the study. Contrast-enhanced CT examinations were acquired at the portal venous phase afterintravenous 2 mL/kg contrast agent at 1–3 mL/s (Omnipaque 350, GE Healthcare, Princeton, NJ, US). Raw image data were reconstructed into contiguous 5 mm axial and coronal slices.

MRI scans of the abdominopelvic region were performed on a 1.5 T MRI scanner (Ingenia, Philips Healthcare, Best, The Netherlands) using two phased-array surface coils. The examination protocol included axial and coronal T2-weighted images, diffusion-weighted images (b = 800 and 1300). Gadolinium-enhanced T1-weighted images with fat suppression were acquired at 4 min following administration of 0.1 mmol/kg Gd-based contrast agent (Omniscan, GE Healthcare, Milwaukee, WI, USA). Sequence parameters are given in Table S1.

Imaging PCI scores were calculated by the same radiologist with 20 years experience in abdominal imaging with free access to patients’ medical records. All images were interpreted preoperatively.

### 2.4. Intraoperative Assessment

The OP-PCI was the reference standard for PCI and was scored by surgeons during surgery. After CRS, the residual tumor was evaluated and the CC score was recorded as CC-0 to CC-3. For patients receiving diagnostic laparotomy but who did not receive CRS due to high PCI or inoperable disease, the postoperative tumor burden was documented as CC-3.

### 2.5. Statistical Analysis

All data are reported as means ± standard deviation (SD). Student t-test was applied for the analysis of the mean difference between different variables. One-way analysis of variance (ANOVA) with Bonferroni post hoc test was used to determine differences between the means of two or more independent groups.The statistical significance level was set at α = 0.05. Correlation and agreement tests were performed using SPSS version 25 (IBM Corp., Armonk, NY, USA). Pearson’s correlation and linear regression analysis were used to examine the relationship between the imaging and intraoperative PCI scores. Pearson correlation coefficient (R) from linear regression was used to test the strength of the linear association. An R of 0.91–1 indicated very high correlation, an R of 0.71–0.90 indicated high correlation, an R of 0.51–0.70 indicated moderate correlation, an R of 0.31–0.50 indicated low correlation, and an R below 0.3 indicated negligible correlation. The standard error of the estimate (SEE) was used to measure the prediction errors of the dataset. The Bland–Altman analysis was used to calculate the bias and limit of agreement (LOA) between methods. Agreement between imaging and intraoperative PCI scores was categorized using intraclass correlation (ICC) with two-way mixed effects and absolute agreement option. An ICC coefficient (r) of 0.81–1.00 indicated almost perfect agreement, an r of 0.61–0.80 indicated substantial agreement, an r of 0.41–0.60 indicated moderate agreement, an r of 0.21–0.40 indicated fair agreement, and an r of 0–0.20 indicated poor agreement.

The final dataset consisted of basic patient information, image findings, and surgical assessment. Clinical data included patient’s age, sex, and primary origin of their peritoneal metastasis. Radiological features included the presence or absence of ascites and fixation of intestinal loops. PCI scores in region 0 to region 12, in all 13 abdominopelvic regions, and regions 9–12 were calculated using CT, and MRI during surgery. Surgical outcomes were divided as complete cytoreduction (CC-0) or incomplete cytoreduction (non-CC-0). Decision tree algorithms, J48 and REPTree, were applied to classify CC-0 vs. non-CC-0 using Weka 3.8.3 [15]. Attributes in the CT subset included clinical and CT data. The MRI subset was constructed with the patient’s clinical data as well as MRI findings. The surgery subset was constructed with clinical data and OP-PCI scores. Learner-based feature selection, the WrapperSubsetEval technique with the GreedyStepwise search method, was used to select input attributes. Five-fold cross-validation was used to split the dataset into training and validation subsets. Evaluation metrics including accuracy, sensitivity, specificity, precision, recall, F-measure, and area under the receiver operating characteristic curve (ROC area) were obtained.

## 3. Results

### 3.1. Patients’ Characteristics

The final analysis included 20 males (56 ± 9 years) and 42 females (56 ± 12 years) with peritoneal metastasis from appendiceal (*n* = 6), colon (*n* = 25), ovarian (*n* = 20), and gastric (*n* = 11) origins. Baseline demographics are presented in Table 1. No significant difference was observed in age between patients with four different types of cancer. Among the 62 patients, the mean duration between CT and surgery was 32 ± 28 days whereas the mean duration between MRI and surgery was 13 ± 17 days. Mean OP-PCI was 15 ± 11 points for all cancer types. Mean PCI in patients with appendiceal cancer was significantly higher than those in patients with the other three cancer types by intraoperative assessment. CC-0 was achieved in 33 (53.2%) patients. Mean PCI scores were significantly higher in patients with non-CC-0 than in those with CC-0 by intraoperative (23 ± 10 vs. 8 ± 6, *p* < 0.001), CT (13 ± 12 vs. 6 ± 5, *p* = 0.002), and MRI (17 ± 10 vs. 6 ± 5, *p* < 0.001) assessments.

### 3.2. Imaging and Intraoperative PCI

The correlation and agreement between imaging PCI and OP-PCI are shown in Table 2. In gastric cancer patients, a large discrepancy (bias = −8.3 points) and poor agreement (ICC = 0.144) with only moderate correlation were appreciated between CT-PCI and OP-PCI. Compared with CT, a smaller bias (−4.6 points) with fair agreement (ICC = 0.407) was noted between MRI-PCI and OP-PCI, suggesting that MRI may be a better imaging modality for preoperative PCI assessment for gastric cancer patients.

In patients with the other three cancer types, imaging methods underestimated PCI by 3.0 to 5.3 points, but there was a high to a very high correlation between imaging and surgical PCI (R = 0.745 to 0.929). In appendiceal cancer patients, the PCI scores obtained by two imaging methods and intraoperative assessment were not significantly different (*p* > 0.05, Table 1), indicating that CT and MRI may have similar performance for predicting PCI. In ovarian cancer patients, preoperative image PCI scores by both CT and MRI were significantly lower than the OP-PCI scores. However, similar degrees of correlation and agreement was noted for PCI scores between both imaging methods and intraoperative assessment. Therefore, there may be a limited role for MRI compared with CT in the assessment of imaging PCI for ovarian cancer patients.

In colon cancer patients, a greater bias and limit of agreement for PCI score was appreciated using CT (bias = −4.9 and LOA = −20.0 to 10.2) compared with MRI (bias = −3.3 and LOA = −13.2 to 6.6). Linear regression analysis showed a very high correlation (R = 0.929) with an SEE of 4.4 points between MRI-PCI and OP-PCI, whereas there was a weaker correlation (R = 0.745) and larger SEE (7.9 points) between CT-PCI and OP-PCI. Moreover, the agreement between MRI-PCI and OP-PCI was almost perfect (ICC = 0.830), while the agreement was substantial between CT-PCI and OP-PCI scores (ICC = 0.646). The above results suggested that MRI may be a more suitable approach than CT for PCI estimation in colon cancer patients.

### 3.3. Model Performance for Predicting Incomplete Cytoreduction

J48 and REPTree algorithms were used for classifying and predicting the surgical outcome (CC-0 or non-CC-0) for patients who underwent CRS/HIPEC. The evaluation metrics for predicting incomplete cytoreduction are shown in Table 3. For the CT dataset, J48 and REPTree produced the same model with identical performance (accuracy = 69.4%, ROC area = 0.635). For the MRI dataset, the REPTree algorithm produced a simpler and more accurate model compared with J48 (accuracy = 79.0% and size of the tree = 9 for REPTree vs. accuracy = 72.6% and size of the tree = 11 for J48). For the surgery model, J48 performed better than REPTree in terms of accuracy of classification, but for the complexity of tree structure, REPTree was better because it generated a simple tree structure (size of tree = 3) while J48 generated a tree with a tree size of 11. The best surgery model generated by REPTree consisted of one root node and two leaf nodes with a branch (PCI ≤ 20 or PCI > 20).

### 3.4. Classifier Models for Predicting Incomplete Cytoreduction

**Figure 1:**
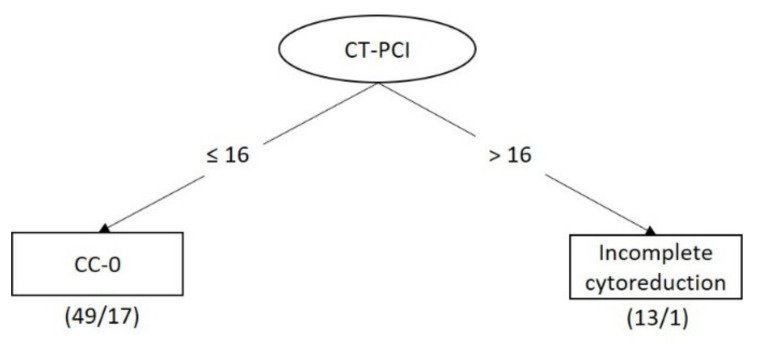
Decision tree for predicting surgical outcome based on clinical data and CT findings. Each node represents a decision rule that splits the data. Terminal nodes correspond to the two classes, complete and incomplete cytoreduction. The first number in the blank is the total number of instances reaching the terminal node. The second number is the number of those instances that are misclassified. Abbreviations: CC-0, complete cytoreduction; CT-PCI, peritoneal cancer index obtained by CT.

Figure 1 shows the best tree structure generated by J48 and REPTree for classifying and predicting incomplete cytoreduction, with a sensitivity of 0.414, a specificity of 0.939, an F-measure of 0.558, and an area under an ROC curve of 0.635 (Table 3). With a CT-PCI > 16, 92.3% (12/13) of the patients could not achieve CC-0.

**Figure 2:**
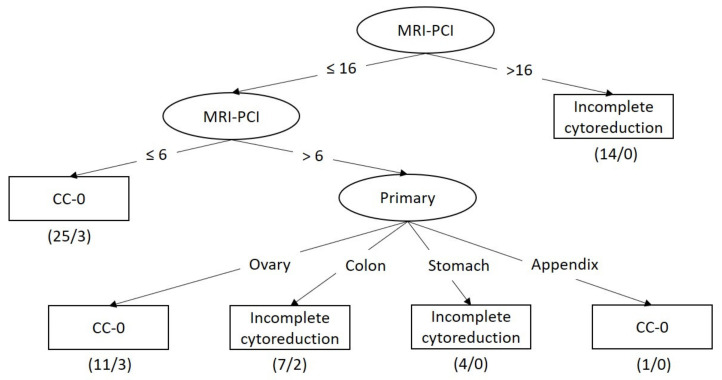
Decision tree for predicting surgical outcome based on clinical data and MRI findings.

Figure 2 shows the best tree structure generated by REPTree, with a sensitivity of 0.690, a specificity of 0.879, an F-measure of 0.755, and an area under a ROC curve of 0.786 (Table 3). With an MRI-PCI >16, all patients could not achieve CC-0. Indeed, the model performance was much improved with MRI features entered into the dataset.

**Figure 3:**
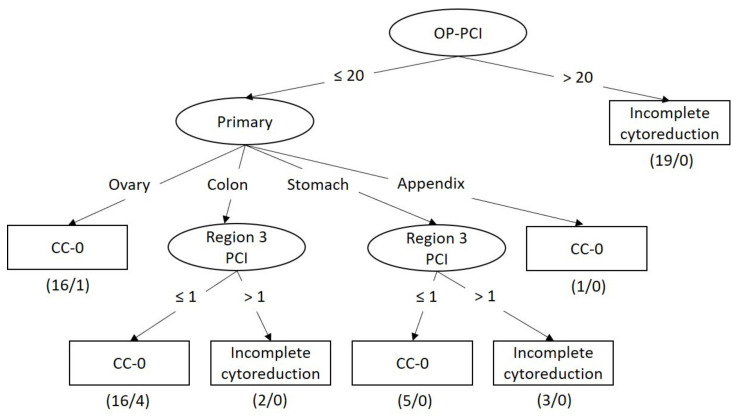
Decision tree for predicting surgical outcome based on clinical data and intraoperative findings. OP-PCI: intraoperative PCI.

Diagnostic or exploratory laparotomy is considered as the best method to assess the PCI and resectability. As expected, the surgery model was considered the best among the three models. The best tree structure for the surgery dataset was generated by J48 (Figure 3), with a sensitivity of 0.793, a specificity of 0.939, an F-measure of 0.852, and an area under the ROC curve of 0.868 (Table 3). With an OP-PCI > 20, none of the patients could achieve CC-0 status.

## 4. Discussion

Preoperative imaging PCI is regarded as a key prognostic factor for CRS/HIPEC and it challenges the radiologist in ensuring an accurate estimation of imaging PCI and identifying the need for further imaging studies in the multidisciplinary team meeting. This prospective study aimed at assessing the value of MRI in addition to CT in the estimation of intraoperative PCI and prediction of surgical outcome for preoperative evaluation of CRS/HIPEC based solely on preoperative information. In the present study, MRI was superior to CT with a stronger correlation and agreement in estimating OP-PCI in both colon cancer and gastric cancer patients but had limited value in appendiceal cancer and ovarian cancer patients. Based on these results, further MRI in addition to CT was suggested for colon and gastric cancer patients, but not for appendiceal and ovarian cancer patients, during preoperative CRS/HIPEC evaluation. For the prediction of surgical outcome, the prediction model based on CT-PCI was not accurate enough to be useful, whereas the MRI model reached an acceptable accuracy (79.0%) and fair discriminatory power (ROC area = 0.786).

Contrast-enhanced CT is generally considered as the preferred first-line diagnostic imaging modality for detecting peritoneal metastasis in clinical practice considering its low cost and fast scanning time. Compared to CT, the use of MRI can improve the sensitivity for detecting small peritoneal tumors owing to its ability to provide different soft tissue contrast by modifying the imaging sequences and parameters [16,17]. However, MRI is sensitive to motion artifacts and the MRI image quality may degrade in patients with poor breath-hold capability and contraindications to antiperistaltic agents. In contrast, CT images can be acquired at a relatively short scan time and are less sensitive to the patient’s movement during the examinations. In a systemic review and meta-analysis study [18], CT was recommended as the preferred imaging modality and MRI or PET/CT as the second choice. However, their data were from patients with various types of cancer, and their suggestions were based on the pooled results. This prospective study recruited patients with peritoneal metastasis from different primary cancer origins for both CT and MRI studies and therefore, it could provide the information about when to arrange MRI scans in addition to CT for patients with different cancer origins to help with the assessment of PCI and surgical outcome.

Very few studies have compared the performance of CT-PCI and MRI-PCI for patients with peritoneal metastasis from a specific type of cancer. In patients with colon cancer, Lee et al. [17] reported a fair agreement between CT-PCI and OP-PCI (ICC = 0.359) and an almost perfect agreement between MRI-PCI and OP-PCI (ICC = 0.854). Similar results were reported by van ‘t Sant et al. [19], who reported a fair to moderate agreement between CT-PCI and OP-PCI (ICC = 0.39 to 0.44) and an almost perfect agreement between MRI-PCI and OP-PCI (ICC = 0.83 to 0.88). In line with previous studies [17,19,20], this study described a better correlation and agreement between MRI-PCI and OP-PCI than between CT-PCI and OP-PCI, suggesting that MRI was superior to CT for colon cancer patients who underwent preoperative CRS/HIPEC assessment, although a better degree of correlation between CT and surgery (ICC = 0.646) was presented in the present study. For patients with peritoneal carcinomatosis from ovarian cancer, the results are controversial. Ahmed et al. [21] reported CT was a suitable tool for predicting PCI in ovarian cancer patients, although another report has shown that MRI was superior to CT [22]. This study showed that MRI was superior to CT in predicting PCI, but the improvement might not be of clinical importance.

The present study showed a high correlation and agreement in PCI scores between both CT and MRI with surgical PCI. However, there were only six patients with peritoneal metastasis from appendiceal cancer, such that the patients could not be divided into invasive and noninvasive subtypes. In a previous study, a similar degree of agreement was noted between CT-PCI and MRI-PCI with OP-PCI (ICC = 0.751–0.753) in noninvasive appendiceal cancer patients while a higher degree of agreement between MRI-PCI and OP-PCI (ICC = 0.822) than between CT-PCI and OP-PCI (ICC = 0.594) in invasive appendiceal cancer patients [17]. Therefore, MRI may be recommended for invasive appendiceal cancer patients, which may be indicated during the preoperative assessment for CRS/HIPEC. However, it is not always feasible to obtain tissue for the preoperative histological subtype in appendiceal cancer patients, and a general recommendation for all the subtypes of appendiceal cancer is still required.

Accurate prediction for patients with inoperable tumor is vital for appropriately selection of patients with peritoneal metastasis for neoadjuvant therapy. The CT model of this study yielded an accuracy of 69.4% and an ROC area of 0.635, indicating a poor discrimination ability for differentiating the result of cytoreduction status. Similar results have been proposed by Liaos et al. [23], with an accuracy of 66% for predicting complete cytoreduction of advanced ovarian cancer patients using nearest-neighbor models with attributes including clinical data and CT findings. Other studies showed a low sensitivity (27%) but high specificity (91%) for predicting incomplete or suboptimal cytoreduction using CT-PCI.

With MRI attributes in the model, the model performance improved, with prediction accuracy reached 79.0% with an ROC area of 0.786, yielding acceptable accuracy for predicting the feasibility of incomplete cytoreduction [24]. In the present study, all patients with an MRI-PCI > 16 (14/14) received non-CC-0, while the cut-off value was higher in the OP model, which showed all patients with an OP-PCI > 20 (19/19) received non-CC-0. An OP-PCI > 20 is commonly used as an exclusion criterion when selecting patients for CRS/HIPEC, and our results agree with the findings. In addition to OP-PCI > 20, this study also suggested that MRI-PCI > 16 may be considered as a contraindication for CRS/HIPEC.

The present study has the strength of providing correlation and agreement between imaging and surgical PCI estimates. Furthermore, an effective MRI model and OP model for incomplete cytoreduction using decision trees with cross-validation were established. The REPTree and J48 tree algorithms were used to predict CC-0 vs. non-CC-0, as decision tree models are more convenient to develop and implement than other machine learning algorithms and may aid in decision making during CRS/HIPEC. This study has the limitations of using a small study population for appendiceal cancer patients. Another limitation is that this is a single-institutional study, causing a selection bias. Furthermore, this study only included basic patient’s age, gender, and cancer type as potential attributes, but not clinical data, which have been reported to be related to surgical outcomes, such as performance status, tumor marker, and histology subtypes. However, this study is still meaningful as it demonstrated different degrees of correlation and agreement between imaging and intraoperative PCI through a prospectively study design.

## 5. Conclusions

CT was suitable for estimating PCI in patients with peritoneal metastasis from appendiceal and ovarian cancer, while further MRI in addition to CT was recommended for patients with peritoneal metastasis from colon and gastric cancer. For classifying patients with peritoneal carcinomatosis into complete and incomplete cytoreduction, MRI was more effective than CT. Multidisciplinary team discussions between surgical oncologists and radiologist will help to optimize patient selection for CRS/HIPEC, but more resources are needed to aid diagnostic and treatment decisions. Future investigations are needed, especially for optimal imaging options for peritoneal metastasis with different cancer stages and grades.

## Figures and Tables

**Table 1:** Baseline demographic characteristics of cancer patients.

	All	Appendix	Colon	Ovary	Stomach	ANOVA
No	62	6	25	20	11	
Sex (M/F)	20/42	3/3	11/14	0/20	6/5	
Age (ys)	56 ± 11	57 ± 13	56 ± 11	55 ± 10	54 ± 13	0.910
**Peritoneal Cancer Index (PCI)**						
Intraoperative	15 ± 11	29 ± 10	13 ± 12	14 ± 10	12 ± 9	0.011
CT	9 ± 10 ***	26 ± 10	8 ± 9 **	9 ± 8 **	4 ± 2 **	<0.001
MRI	11 ± 9 ***	25 ± 9	10 ± 8 **	10 ± 9 **	7 ± 8	0.001
**Cytoreduction Score (** **CC** **)**						
CC-0	33	1	12	15	5	
CC-1	8	2	3	2	1
CC-2	4	1	1	2	0	
CC-3	17	2	9	1	5	

Data are presented as mean ± SD. One-way analysis of variance (ANOVA) was used to test the difference among means of age and peritoneal cancer index among peritoneal cancer of four different origins. Student’s *t*-test was used to test the differences of peritoneal cancer index between CT and MRI with intraoperative assessment. **, *p* < 0.01; ***, *p* < 0.001.

**Table 2:** Correlation and agreement between imaging and intraoperative peritoneal cancer index.

	Linear Regression						Bland-Altman Analysis		
Estimate	R	b	95% CI	Constant	95% CI	SEE	Bias	LOA	ICC (r)
**All**									
CT-PCI	0.775	0.9	0.7, 1.1	6.2	3.7, 8.8	7.1	−5.3	−19.3, 8.6	0.680
MRI-PCI	0.856	1.0	0.9, 1.2	3.6	1.3, 5.9	5.8	−3.8	−15.1, 7.5	0.792
**Appendix**									
CT-PCI	0.851	0.8	0.1, 1.5	7.7	−11.4, 26.8	5.7	−3.0	−13.7, 7.7	0.833
MRI-PCI	0.879	0.9	0.2, 1.7	4.8	−14.2, 23.7	5.2	−3.5	−12.7, 5.7	0.835
**Colon**									
CT-PCI	0.745	1.0	0.6, 1.4	4.8	0.2, 9.4	7.9	−4.9	−20.0, 10.2	0.646
MRI-PCI	0.929	1.3	1.1, 1.6	−0.1	−3.0, 2.8	4.4	−3.3	−13.2, 6.6	0.830
**Ovary**									
CT-PCI	0.781	0.9	0.5, 1.3	5.7	1.4, 10.1	6.2	−4.9	−16.8, 7.0	0.679
MRI-PCI	0.858	1.0	0.7, 1.2	4.5	0.8, 8.1	5.1	−4.1	−13.8, 5.6	0.782
**Stomach**									
CT-PCI	0.522	2.1	−0.5, 4.7	4.0	−7.4, 15.5	8.3	−8.3	−24.4, 7.9	0.144
MRI-PCI	0.450	0.5	−0.3, 1.3	8.2	0, 16.5	8.7	−4.6	−22.4, 13.2	0.407

Abbreviations: PCI, peritoneal cancer index; R, Pearson’s correlation coefficient; B, Constant, coefficients in linear regression model: Estimate = b(B) + Constant; CI, confidence interval; SEE, standard error of the estimate; LOA, limits of agreement; ICC, intraclass correlation; r, ICC coefficient.

**Table 3:** Model evaluation metrics for predicting incomplete cytoreduction using tree algorithms.

	Tree Size	Accuracy	Sensitivity	Specificity	Precision	Recall	F-Measure	ROC Area
**CT Model**								
J48	3	69.4%	0.414	0.939	0.857	0.414	0.558	0.635
REPTree	3	69.4%	0.414	0.939	0.857	0.414	0.558	0.635
**MRI Model**								
J48	11	72.6%	0.690	0.758	0.714	0.690	0.702	0.749
REPTree	9	79.0%	0.690	0.879	0.833	0.690	0.755	0.786
**OP Model**								
J48	11	87.1%	0.793	0.939	0.920	0.793	0.852	0.868
REPTree	3	82.3%	0.655	0.970	0.950	0.655	0.776	0.783

Abbreviations: ROC area, area under receiver operating characteristics curve; OP, operation.

## Data Availability

The data presented in this study are available on request from the corresponding author.

## Supplementary Materials

The following are available online at https://www.mdpi.com/article/10.3390/diagnostics11040674/s1, Table S1: Examination of MRI protocol and sequence parameters.
