# Supplementary material for: Adding Value of MRI over CT in Predicting Peritoneal Cancer Index and Completeness of Cytoreduction
Source: Diagnostics (Basel). 2021 Apr 8;11(4):674. doi: 10.3390/diagnostics11040674 (PMC8068380; doi:10.3390/diagnostics11040674)
Supplement: Supplementary file 1 [file diagnostics-11-00674-s001.pdf]

Supplementary Materials

Adding Value of MRI Over CT in Predicting Peritoneal Cancer Index and Completeness of Cytoreduction

Chia-Ni Lin [1], Weh-Shih Huang [2], Tzu-Hao Huang [3], Chao-Yu Chen [4], Cheng-Yi Huang [2], Ting-Yao Wang [5], Yu-San Liao [1] and Li-Wen Lee [1,*]

1 Department of Diagnostic Radiology, Chang Gung Memorial Hospital, Chiayi 613016, Taiwan; lcn6979@cgmh.org.tw (C.-N.L.); mm601200@gmail.com (Y.-S.L.)
2 Division of Colorectal Surgery, Department of Surgery, Chang Gung Memorial Hospital, Chiayi 613016, Taiwan; wen1204@cgmh.org.tw (W.-S.H.); bluesky@cgmh.org.tw (C.-Y.H.)
3 Division of General Surgery, Department of Surgery, Chang Gung Memorial Hospital, Chiayi 613016, Taiwan; tambobo8916@gmail.com
4 Department of Obstetrics and Gynecology, Chang Gung Memorial Hospital, Chiayi 613016, Taiwan; b9002031@cgmh.org.tw
5 Division of Hematology and Oncology, Department of Internal Medicine, Chang Gung Memorial Hospital, Chiayi 613016, Taiwan; tywang.onco@gmail.com
* Correspondence: m4572@cgmh.org.tw; Tel.: +886 5 3621 000

Table S1. Examination of MRI protocol and sequence parameters.

	T 2WI	T2WI	T2WI	DWI	T1+C
Sequence	SE	SE	SE	SE	SE
TR (msec)	700	4300	1000	4300	4
TE (msec)	60	100	70	48	2
Matrix	220×156	384×226	252×2209	100×98	332×251
Slice (mm)	5	5	5	6	5
Gap (mm)	1	1.5	1	0	0
FOV (cm)	35	35	40	40	40
Voxels (mm)	1.7×1.9×5	0.9×1.5×5	1.6×1.9×5	4×4.1×6	1.2×1.2×5
Flip angle	90°	90°	90°	90°	10°
BW (Hz/pixel)	650	260	636	3969	433
Average	1	2	1	1	1
Fat suppressed	No	No	No	Yes	Yes
Plane	Axial	Axial	Coronal	Axial	Axial
Diffusion b-value	0	0	0	800, 1300	0
Notes	Abdomen	Pelvis	Abdomen + Pelvis	Abdomen + Pelvis	Abdomen + Pelvis
